# Adherence to the cervical cancer screening program in women living with HIV in Denmark: comparison with the general population

**DOI:** 10.1186/1471-2334-14-256

**Published:** 2014-05-13

**Authors:** Kristina Thorsteinsson, Steen Ladelund, Søren Jensen-Fangel, Terese L Katzenstein, Isik Somuncu Johansen, Gitte Pedersen, Jette Junge, Marie Helleberg, Merete Storgaard, Anne-Mette Lebech

**Affiliations:** 1 Department of Infectious Diseases, Hvidovre Hospital, Copenhagen University Hospital, Kettegaards Allé 30, DK-2650 Hvidovre, Denmark; 2 Clinical Research Center, Hvidovre, Copenhagen University Hospital, Copenhagen, Denmark; 3 Department of Infectious Diseases, Skejby, Aarhus University Hospital, Skejby, Denmark; 4 Department of Infectious Diseases, The National University Hospital, Rigshospitalet, Copenhagen, Denmark; 5 Department of Infectious Diseases, Odense University Hospital, Odense, Denmark; 6 Department of Infectious Diseases, Aalborg University Hospital, Aalborg, Denmark; 7 Department of Pathology, Hvidovre, Copenhagen University Hospital, Hvidovre, Denmark

**Keywords:** Cervical, Cancer, Screening, HIV, Adherence, Attendance, Guidelines

## Abstract

**Background:**

Women living with HIV (WLWH) are at increased risk of invasive cervical cancer (ICC). International HIV guidelines suggest cervical screening twice the first year after HIV diagnosis and thereafter annually. Adherence to the HIV cervical screening program in Denmark is unknown.

**Methods:**

We studied women from a population-based, nationwide HIV cohort in Denmark and a cohort of age-matched females from the general population. Screening behaviour was assessed from 1999–2010. Adjusted odds ratios (OR’s) for screening attendance in the two cohorts and potential predictors of attendance to guidelines were estimated. Pathology specimens were identified from The Danish Pathology Data Bank.

**Results:**

We followed 1143 WLWH and 17,145 controls with no prior history of ICC for 9,509 and 157,362 person-years. The first year after HIV diagnosis 2.6% of WLWH obtained the recommended two cervical cytologies. During the different calendar intervals throughout the study period between 29-46% of WLWH followed the HIV cervical screening guidelines. Adjusted OR’s of attendance to the general population screening program for WLWH aged 30, 40 and 50 years, compared to controls, were 0.69 (95% CI: 0.56-0.87), 0.67 (0.55-0.80) and 0.84 (0.61-1.15). Predictors of attendance to the HIV cervical screening program were a CD4 count > 350 cells/μL and HIV RNA < 500 copies/mL. Calendar period after 2002 and HIV RNA < 500 copies/mL predicted attendance to the general population cervical screening program.

**Conclusions:**

The majority of WLWH do not follow the HIV guidelines for cervical screening. We support the idea of cytology as part of an annual review and integration of HIV care and cervical screening in a single clinic setting.

## Background

Invasive cervical cancer (ICC) is the second most common cancer in women worldwide [1,2]. Women living with HIV (WLWH) have an increased prevalence of Human Papillomavirus (HPV) infection [1,3-5], which is the main cause of ICC [1,3,6-8]. Furthermore, an increased risk of progression to cervical dysplasia and ICC is reported in WLWH compared to non-HIV-infected peers [1,3,7-11]. As a consequence international guidelines suggest an intensified screening program for ICC in WLWH with annual visits [1]. Whereas for women living without HIV in Denmark, screening is recommended every third year in women aged 23–49 years and every fifth year in women aged 50–65 years [12]. However, data on screening attendance is scarce and studies only cover short time-spans and predominantly rely on data, where patients in retrospect report their screening habits [13-17].

With timely and appropriate screening and treatment of pre-invasive lesions, ICC is highly preventable [1,5,7,18,19]. The introduction of screening programs for ICC has led to a notable decline in ICC incidence in the general population [5,18,19], but non-attendance to screening programs constitutes a major problem [19].

Denmark is a unique setting to study screening behaviour. Due to the Danish Civil Personal Registration number (CPR) [20] we are able to link databases with complete, nationwide information ranging from data on date of birth/death, HIV status, pathology specimens, hospital diagnoses etc.

We aimed to examine the cervical screening coverage in WLWH in Denmark compared to population controls without HIV infection and to identify predictors for attendance to the screening program.

## Methods

### Ethics statement

The study was approved by the Danish Data Protection Agency (2010-331-0468 and 2012-331-0082) and the Danish HIV Cohort Study (DHCS) is approved by the Danish Data Protection Agency (2008-41-1781). Ethics approval and individual consent are not required by Danish legislation governing this type of study (“Act on Research Ethics Review of Health Research Projects”, June 14, 2001, Section 10) [21].

### Setting

Denmark has a population of 5.6 million [22] and an estimated HIV prevalence among adults of 0.1% [23]. Medical care, including highly active antiretroviral therapy (HAART), is tax-paid and provided free-of-charge to individuals living with HIV in Denmark. Treatment of HIV is restricted to eight specialized medical centres, where patients are seen on an outpatient basis at intended intervals of 12–24 weeks.

### Cervical screening in Denmark

A population-based screening program for ICC was introduced in Denmark in the mid-1960s. The Danish National Board of Health now recommend that women aged 23–49 years receive personal invitations for screening every third year and women aged 50–65 years every fifth year [24]. If a cervical cytology is not received a reminder is sent out after 3 months and again after 6 months. Screening is free of charge and most often the general practitioner is responsible for sample taking. Women immigrating to Denmark get an invitation for cervical screening as soon as they receive a personal identification number (PIN) (see below). Danish guidelines do not recommend cervical screening after a total hysterectomy for benign disease or during pregnancy, but screening can be resumed 8–12 weeks postpartum [25].

A special cervical screening program in WLWH (HIV screening program) has been recommended since 1995, twice the first year after HIV diagnosis and annually thereafter (target age group has not been specified) [1]. In this setting annual written invitations are not implemented and screening relies on information from health care professionals to WLWH. Pregnant WLWH should have cervical cytology at their initial prenatal visit unless a normal cervical cytology has been obtained within the past year [1].

### Registries the civil registration system

The CRS is a national registry of all Danish residents containing information on date of birth, date of migration and date of death [20]. At birth or immigration a 10-digit PIN (CPR) is assigned to each individual, which allows accurate linkage between population-based registries and enables treatment centres to avoid multiple registrations of the same patient. Population controls for this study were identified from the CRS. We used the CPR to link data from the following registers:

### Danish HIV cohort study

The DHCS is a prospective, observational, nationwide, multicentre, population-based

cohort study of all individuals living with HIV seen at the Danish HIV clinics since 1 January 1995. The cohort has been described in detail elsewhere [26]. In brief, data collection is ongoing, with continuous enrolment of both newly diagnosed residents and immigrants with HIV. Data is updated annually and among other variables contains: gender, date of HIV infection, date of death and HAART regimen. Laboratory data include CD4 counts and HIV RNA.

### The Danish Pathology Data Bank (DPDB)

The DPDB was established in 1999 and contains detailed nationwide records of all pathology specimens analyzed in Denmark since 1997 [27]. Data on cytology was retrieved using the Systemized Nomenclature of Medicine (SNOMED) code of cervix uteri: T8×3*.

### The National Patient Registry (NPR)

The NPR contains records of all inpatient hospital diagnoses since 1977 and outpatient hospital diagnoses since 1995 [28]. To assess pregnancy we used the ICD-10 codes O60.9 and O80.0-O84.9 for births.

### The Danish Cancer Registry (DCR)

The DCR is a population-based register and contains information on all incident cancers diagnosed in Danish citizens since 1943 [29]. Diagnoses of prior ICC were obtained using the ICD-10 codes C53.0 - C53.9.

### Study population

#### HIV cohort

We identified all WLWH from the DHCS with a Danish PIN and > 16 years of age at time of HIV diagnosis. The indexdate was defined as 1 January 1999, date of HIV diagnosis, date of 18^th^ birthday or date of immigration, whichever came last. WLWH with a history of ICC or carcinoma in situ prior to indexdate were excluded.

#### Population controls

For each WLWH we identified 15 age-matched women without known HIV from the general population in the CRS who were alive on the patient’s indexdate. Population controls were assigned the same indexdate as the WLWH to whom they were matched. Only women with no history of ICC or carcinoma in situ prior to indexdate were included.

### Statistical analyses

The study period ran from 1 January 1999 until 31 December 2010. In all analyses of screening we studied women from age 23–65 years. To allow for a short period of patient’s/doctor’s delay we added a grace period of 3 months to all screening intervals. Hence, the first year after HIV diagnosis equalled 15 months after HIV diagnosis. Women aged 23–49 years were considered covered by the general population screening program three years plus a 3-month grace period after a cervical cytology and five years plus a 3-month grace period for women aged 50–65 years. WLWH were considered covered by the HIV screening program for one year plus a 3-month grace period independently of age.

When studying the HIV screening program for cervical cancer the first year after HIV diagnosis a WLWH fulfilled screening criteria if she had obtained two cervical cytologies within 15 months and they were at least four months apart to reflect two screening courses. Women infected perinatally or diagnosed with HIV before immigration to Denmark were not included in this analysis.

To study screening attendance during follow-up we evaluated the coverage for each woman at the beginning of every calendar month in the follow-up period. Women in the two cohorts were evaluated in four categories: i) Controls, according to the general population screening program; ii) WLWH, according to the general population screening program; iii) WLWH, before HIV diagnosis, according to the general population screening program; iv) WLWH, according to the HIV screening program.

We estimated age specific Odds ratios (OR’s) of predictors for attendance to the two screening programs at different ages by taking the month observed closest to age 30, 40 and 50 years (+/- 3 years) and determined whether or not the woman was covered by the screening program.

Plots on screening attendance were produced plotting screening attendance against calendar time and age (in years), respectively. The plot representing attendance plotted up against age was ragged and subsequently smoothed.

Women were not included in the analyses during pregnancy (defined as 37 weeks before delivery, and 6 months postpartum) and after hysterectomy (defined as censorship from the date of surgery). Women immigrating to Denmark had a 3-month grace period before they were included in the screening analyses. Lastly, since pathology data was available from 1999, we initiated the observation of screening attendance in 2002 and 2004, to allow for a 3- and 5-year observation period before the indexdate for women aged 23–49 and 50–65, respectively, to see if they were covered by screening according to the general population screening program.

The OR’s and 95% confidence intervals (CI) for predictors of screening were estimated in multivariate logistic regression analyses and adjusted for calendar period when comparing WLWH and controls.

In the analyses of coverage of the general population screening program and HIV screening program in WLWH two models were computed, since CD4 count and HIV RNA are dependent covariates and could not be included in the same model. Route of infection, ethnicity and calendar period were included in both models, whereas time-updated HIV RNA was included in the first model and replaced by CD4 count in the second model. We only presented the OR of the CD4 count from the second model.

Individuals with missing explanatory values were excluded from multivariate regression analyses. The validity of the model was tested using the Hosmer and Lemeshow Goodness-of-Fit Test.

Undetectable viral load was defined as a plasma HIV RNA load of < 500 copies/mL, which was the highest level of sensitivity for testing in the observation period. Significance level was set at 0.05 (two-sided).

SAS statistical software version 9.2 (SAS Institute Inc., Cary, NC, USA) was used for data analysis.

## Results

From the DHCS we identified 1172 WLWH fulfilling the inclusion criteria. Of these, 29 had a prior history of ICC or carcinoma in situ and were excluded from the analyses. Thus leaving 1143 WLWH and 17,145 age-matched female controls in the study, representing a total of 9,509 and 157,362 person-years of follow-up. Characteristics of the patients and controls are described in Table 1.

**Table 1:** Characteristics of women living with HIV and controls

	**Women living with HIV**	**Controls**
Number of individuals	1143	17,145
Follow-up (years), median (IQR)	9.4 (5.0-12.0)	10.8 (6.6-12.0)
Follow-up time, total (person-years)	9,509	157,362
Age at inclusion (years), median (IQR)	33.7 (29.0-40.1)	33.7 (29.0-40.1)
Race, n (%)		
White	548 (47.9)	-^1^
Asian	122 (10.7)	
Black	424 (37.1)	
Other	31 (2.7)	
Missing	18 (1.6)	
Place of HIV transmission^2^, n (%)		
Denmark	436 (38.2)	-^1^
Europe + US	73 (6.4)	
Africa	387 (33.9)	
Asia	103 (9.0)	
Other	7 (0.6)	
Missing	137 (12.0)	
Route of infection, n (%)		
Heterosexual	886 (77.5)	-^1^
IDU	166 (14.5)	
Other	34 (3.0)	
Missing	57(5.0)	
CD4 count at inclusion, (cells/μL),		
< 200	297 (26.0)	-^1^
200–350	288 (25.2)	
> 350	476 (41.6)	
Missing	82 (0.7)	
Hepatitis C co-infection, n (%)		
Yes	238 (20.8)	-^1^
No	905 (79.2)	
AIDS at inclusion, n (%)		
Yes	33 (2.9)	-^1^
No	1110 (97.1)	

^1^No information available, ^2^Self-reported geographic location at the time of HIV acquisition.

### ICC screening according to the HIV screening program

**Figure 1:**
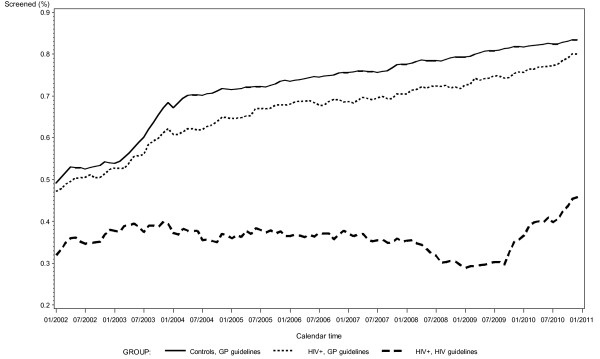
**The proportion of women attending the cervical cancer screening program during the observation period.** Women living with HIV (WLWH) and controls are divided into three categories: i) Controls, studied according to the general population (GP) screening program; ii) WLWH, studied according to the GP screening program; iii) WLWH, studied according to the HIV screening program. Since no data is available before 1999 the curve has been started in 2002 to allow for three years of observation.

During the first year after the HIV diagnosis, 24 (2.6%) of 915 patients eligible for analysis obtained two cervical cytologies (as recommended in the HIV screening program for ICC in WLWH), while 266 (29.1%) had at least one Pap test performed (data not shown). The proportion of WLWH attending screening according to the HIV screening program during the study period remained relatively stable between 29-46% (Figure 1).

### ICC screening according to the general population screening program

Attendance to cervical screening according to the general population program increased during the study period for both WLWH and women in the control group from 47% to 80%, and 49% to 83%, respectively (and from 28% to 57% in the group of women observed before they were diagnosed with HIV infection) (Figure 1). The plot illustrating data on women observed, before they were diagnosed with HIV infection is not shown, due to a limited number of observations.

### Age-related attendance to the general population screening program in WLWH and controls

**Figure 2:**
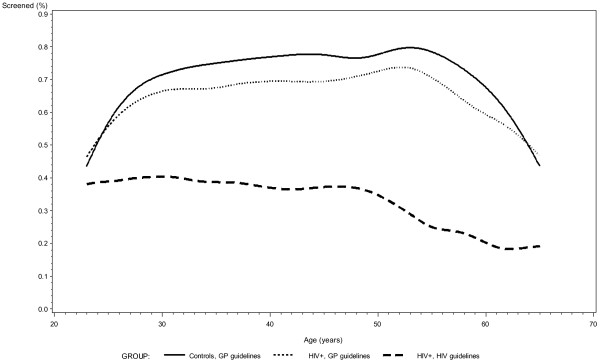
The proportion of women attending the cervical cancer screening program distributed by age divided into three categories: i) Controls, studied according to the general population (GP) screening program; ii) women living with HIV (WLWH), studied according to the GP screening program; iii) WLWH, studied according to the HIV screening program.

When comparing age-related attendance to the general population screening program in WLWH and controls we found that for women aged 30 years, 228 (60.2%) and 4083 (68.4%), respectively (adjusted OR 0.69, 95% CI 0.56-0.87, adjusted p = 0.0011) followed the recommendations, for women aged 40 years, 377 (67.0%) and 6718 (75.0%), respectively followed the recommendations (adjusted OR 0.67, 95% CI 0.55-0.80, adjusted p < 0.0001), and for women aged 50 years 164 (75.2%) and 2943 (78.7%), respectively followed the recommendations (adjusted OR 0.84, 95% CI 0.61-1.15, adjusted p = 0.28) (data on OR’s not shown) (Figure 2).

### Predictors of attendance to the HIV screening program

Predictors of attendance to the HIV screening program in the adjusted analyses in the three age groups were: 30 years: HIV RNA < 500 copies/mL and CD4 count > 200 cells/μL; 40 years: HIV RNA < 500 copies/mL; 50 years: CD4 count > 350 cells/μL as opposed to a CD4 count between 200–350 cells/μL. HIV RNA < 500 copies/mL and a CD4 count > 350 cells/μL were predictors for attendance in all age groups in the unadjusted analyses (Table 2).

**Table 2:** **Predictors of attendance to the HIV cervical cancer screening program in WLWH**^**1 **^**aged 30, 40 and 50 years**

**Predictors of attendance in different age groups**	**30 years (n = 386)**			**40 years (n = 555)**			**50 years (n = 214)**		
	n (%) attending screening	Unadjusted	Adjusted	n (%) attending screening	Unadjusted	Adjusted	n (%) attending screening	Unadjusted	Adjusted
Mode of transmission,									
Heterosexual contact	124 (45.4)	1.00‡	1.00‡	177 (45.9)	1.00‡	1.00‡	72 (50.0)	1.00‡	1.00‡
Intravenous drug use	11 (44.0)	0.94 (0.41-2.15)	0.77 (0.31-1.92)	19 (35.2)	0.64 (0.35-1.16)	0.64 (0.33-1.23)	10 (43.5)	0.77 (0.32-1.87)	0.91 (0.34-2.45)
Other	2 (33.3)	0.60 (0.11-3.35)	0.48 (0.08-2.77)	4 (33.3)	0.59 (0.18-1.99)	0.52 (0.15-1.78)	2 (66.7)	2.00 (0.18-22.55)	1.93 (0.16-22.57)
(missing)	(82)			(103)			(44)		
Race,									
White	51 (48.6)	1.00‡	1.00‡	95 (47.7)	1.00‡	1.00‡	46 (46.5)	1.00‡	1.00‡
Asian	22 (38.6)	0.67 (0.35-1.28)	0.51 (0.25-1.07)	24 (48.0)	0.99 (0.53-1.84)	0.93 (0.48-1.80)	7 (58.3)	1.61 (0.48-5.43)	1.66 (0.42-6.57)
Black	65 (43.9)	0.83 (0.50-1.37)	0.75 (0.43-1.32)	87 (42.2)	1.25 (0.84-1.85)	0.72 (0.47-1.12)	29 (50.0)	1.15 (0.60-2.20)	0.90 (0.43-1.86)
Other	4 (57.1)	1.41 (0.30-6.62)	1.22 (0.25-5.96)	5 (31.3)	2.01 (0.67-5.99)	0.49 (0.14-1.64)	3 (60.0)	1.73 (0.28-10.80)	0.87 (0.11-6.57)
(missing)	(69)			(84)			(40)		
Calendar period									
01.01.2002 – 31.12.2002^2^	47 (37.0)	1.00‡	1.00‡	35	1.00‡	1.00‡	5 (55.6)	1.00‡	1.00‡
01.01.2003 – 31.12.2010	99 (38.2)	1.05 (0.68-1.63)	0.97 (0.59-1.62)	(35.0)176 (38.7)	1.17 (0.75-1.84)	1.00 (0.59-1.67)	81 (39.5)	0.52 (0.14-2.00)	0.39 (0.07-2.11)
Time-updated HIV RNA (copies/mL)									
>500	48 (27.8)	**1.00†**	**1.00†**	43 (26.9)	**1.00†**	**1.00†**	12 (26.1)	**1.00†**	1.00‡
<500	96 (47.3)	**2.34 (1.52-3.60)**	**2.06 (1.27-3.33)**	165 (43.2)	**2.07 (1.38-3.10)**	**1.87 (1.18-2.97)**	73 (45.6)	**2.38 (1.15-4.92)**	2.21 (0.98-4.99)
(missing)	(10)			(13)			(8)		
Time-updated CD4 count (cells/μL)									
< 200	11 (19.3)	**1.00†**	**1.00†**	16 (23.9)	**1.00†**	1.00‡	6 (25.0)	**1.00†**	**1.00†**
200–350	31 (36.9)	**2.45 (1.11-5.41)**	**2.48 (1.06-5.76)**	34 (32.4)	**1.53 (0.76-3.06)**	1.17 (0.55-2.49)	8 (24.2)	**0.96 (0.28-3.25)**	**0.61 (0.16-2.42)**
> 350	102 (43.2)	**3.18 (1.57-6.45)**	**3.02 (1.42-6.42)**	159 (42.9)	**2.39 (1.32-4.35)**	1.58 (0.82-3.05)	71 (47.3)	**2.69 (1.01-7.17)**	**2.41 (0.79-7.33)**
(missing)	(9)			(12)			(7)		

Unadjusted and adjusted odds ratios (OR’s) for predictors of attendance. Predictors of attendance to the cervical screening program in different age groups were estimated including the month observed closest to 30, 40 and 50 years (+/- 3 years) to determine whether or not the woman was covered by screening. Two models are shown in the table: Mode of transmission, ethnicity and calendar period were included in both models, whereas time-updated HIV RNA was included in the first model and replaced by CD4 count in the second model. We only presented the OR of the CD4 count from the second model. ^1^Women living with HIV (WLWH). ^2^Since no data was available before 1999 the reference period only included 2002 to allow for three years observation before initiation.

†The simultaneous test was significant. ‡The simultaneous test was insignificant.

### Predictors of attendance to the general population screening program

Attendance of WLWH to the general population screening program improved after year 2002 for women age 30 and 40 years (adjusted OR 1.77, 95% CI 1.04-3.02 and adjusted OR 3.45, 95% CI 2.05-5.78). Additionally, HIV RNA < 500 copies/mL was a predictor for attendance (Table 2). In the unadjusted analyses predictors of attendance in the three age groups were: 30 years: Calendar period after 2002 and HIV RNA < 500 copies/mL; 40 years: Heterosexual route of infection, calendar period after 2002, HIV RNA < 500 copies/mL and CD4 count > 350 cells/μL; 50 years: HIV RNA < 500 copies/mL (Table 3).

**Table 3:** **Predictors of attendance to the general population cervical cancer screening program in WLWH**^**1 **^**aged 30, 40 and 50 years**

**Predictors of attendance in different age groups**	**30 years (n = 379)**			**40 years (n = 563)**			**50 years (n = 218)**		
	n (%) attending screening	Unadjusted	Adjusted	n (%) attending screening	Unadjusted	Adjusted	n (%) attending screening	Unadjusted	Adjusted
Mode of transmission,									
Heterosexual contact	197 (70.1)	1.00‡	1.00‡	315 (78.6)	**1.00†**	1.00‡	132 (89.2)	1.00‡	1.00‡
Intravenous drug use	15 (60.0)	0.64 (0.28-1.48)	0.53 (0.21-1.35)	36 (63.2)	**0.47 (0.26-0.84)**	0.70 (0.35-1.38)	23 (82.1)	0.56 (0.19-1.67)	0.74 (0.22-2.46)
Other	4 (57.1)	0.57 (0.13-2.60)	0.42 (0.09-2.02)	8 (66.7)	**0.55 (0.16-1.86)**	0.37 (0.11-1.32)	3 (100)	-^2^	-^2^
(missing)	(66)			(93)			(0)		
Race,									
White	76 (71.0)	1.00‡	1.00‡	154 (73.7)	1.00‡	1.00‡	89 (84.8)	1.00‡	1.00‡
Asian	36 (62.1)	0.67 (0.34-1.31)	0.45 (0.21-0.98)	42 (80.8)	1.50 (0.71-3.19)	1.14 (0.51-2.60)	12 (92.3)	2.16 (0.26-17.76)	1.66 (0.19-14.77)
Black	106 (68.8)	0.90 (0.53-1.54)	0.72 (0.38-1.34)	170 (79.8)	1.41 (0.90-2.23)	1.29 (0.77-2.18)	56 (93.3)	2.52 (0.80-7.91)	1.81 (0.52-6.26)
Other	6 (85.7)	2.45 (0.28-21.17)	1.98 (0.22-17.68)	11 (68.8)	0.79 (0.26-2.36)	1.14 (0.32-4.11)	6 (100)	-^1^	-^2^
(missing)	(53)			(73)			(34)		
Calendar period									
01.01.2002 – 31.12.2002^3^	65 (51.2)	**1.00†**	**1.00†**	48 (46.6)	**1.00†**	**1.00†**	8 (72.7)	1.00‡	1.00‡
01.01.2003 – 31.12.2010	163 (64.7)	**1.75 (1.13-2.69)**	**1.77 (1.04-3.02)**	329 (71.5)	**2.88 (1.86-4.45)**	**3.45 (2.05-5.78)**	156 (75.4)	1.15 (0.29-4.49)	1.04 (0.12-9.33)
Time-updated HIV RNA (copies/mL)									
> 500	86 (52.8)	**1.00†**	1.00‡	90 (54.2)	**1.00†**	**1.00†**	29 (60.4)	**1.00†**	1.00‡
< 500	137 (66.8)	**1.80 (1.18-2.75)**	1.37 (0.83-2.28)	279 (73.0)	**2.29 (1.57-3.34)**	**1.76 (1.08-2.88)**	130 (79.8)	**2.58 (1.29-5.16)**	2.43 (0.88-6.71)
(missing)	(11)			(15)			(7)		
Time-updated CD4 count (cells/μL)									
< 200	33 (56.9)	1.00‡	1.00‡	40 (56.3)	**1.00†**	1.00‡	14 (58.3)	1.00‡	1.00‡
200–350	42 (52.5)	0.84 (0.42-1.65)	0.49 (0.21-1.13)	61 (58.7)	**1.10 (0.60-2.02)**	0.67 (0.30-1.51)	24 (70.6)	1.71 (0.57-5.13)	1.54 (0.36-6.53)
> 350	147 (63.9)	1.34 (0.75-2.41)	0.76 (0.36-1.61)	269 (71.9)	**2.00 (1.18-3.34)**	1.07 (0.52-2.19)	121 (79.1)	2.70 (1.10-6.64)	4.33 (1.24-15.10)
(missing)	(11)			(14)			(7)		

Unadjusted and adjusted odds ratios (OR’s) for predictors of attendance. Predictors of attendance to the general population cervical screening program in different age groups were estimated including the month observed closest to 30, 40 and 50 years (+/- 3 years) to determine whether or not the woman was covered by screening. Two models are shown in the table: Mode of transmission, ethnicity and calendar period were included in both models, whereas time-updated HIV RNA was included in the first model and replaced by CD4 count in the second model. We only presented the OR of the CD4 count from the second model, ^1^Women living with HIV (WLWH), ^2^Could not be estimated, due to shortage of events, ^3^Since no data was available before 1999 the reference period only included 2002 to allow for three years observation before initiation.

†The simultaneous test was significant. ‡The simultaneous test was insignificant.

In all adjusted analyses we performed sensitivity analyses to check for the effect of missing values on outcome by adding an extra category with missing values. However, this had no impact on the estimates.

## Discussion

We found that between 29-46% of WLWH followed the recommended HIV cervical screening program during the study period. Moreover, WLWH displayed lower attendance to the less demanding general population screening program for ICC than controls. Screening attendance to the general population screening program improved gradually after 2002, but not according to the HIV screening program and a predictor of attendance to both guidelines was HIV RNA < 500 copies/mL. Lastly, a CD4 count > 350 cells/μL predicted attendance to the HIV screening program.

Attendance to the HIV cervical screening program the first year after HIV diagnosis was remarkably low with only 2.6% of WLWH attending the two recommended cytologies. This might reflect that during the first year after HIV diagnosis focus is on other urgent physical and mental health issues.

We report a cervical screening coverage between 60% and 75% depending on age in WLWH according to the general population screening program and a screening coverage during the study period on only about a third to a half of all WLWH according to the HIV cervical screening program. The screening coverage found in the present study is considerably lower than what have been published in previous studies reporting a coverage to the annual ICC HIV screening program between 51% and 81% [13,14,16,30]. These studies are based on self-reported data, and the difference might reflect that women tend to over-report their participation in cervical screening in a given timeframe and therefore these rates are likely upper-end estimates [31]. Furthermore, unlike register-based studies, to participate in studies based on self-reported data, women being recruited have to attend care for HIV infection else they are excluded a priori. In line with this, data concerning the general population suggest that women are less likely to have received a cervical cytology when they have no contact to primary care [32]. The Swiss HIV Cohort Study found a self-reported semi-annual coverage of 35% [17], but studies are hard to compare due to the study design with semi-annual questionnaires.

Two register-based studies from New Zealand and the UK both found a relatively high coverage of cervical screening of 68% and 74%, respectively, within the previous 12 months [33,34]. However, in New Zealand the annual review for WLWH included the option of having cervical cytology performed at the sexual health clinic when attending for HIV-related care. A small Australian audit found a screening coverage comparable to the present study of only 27% the previous year [15], which the authors among others ascribe cultural reticence in immigrants and lack of health knowledge. In a postnatal cohort from Ukraine 30% of women had a cervical cytology performed in the past, though only tests taken as a part of HIV care were registered [7].

In the present study both WLWH and controls were more likely to take up cervical screening according to the general population screening program after 2002, probably owing to more focus on cancer screening programs per se in the Danish society. This did however not translate into increased screening attendance in WLWH according to the HIV cervical screening program.

In line with Shah et al. [35] we found that an HIV RNA < 500 copies/mL as a proxy for being on HAART and therefore attending the HIV clinics for regular controls and delivery of free HAART was a predictor for screening attendance. This difference was only found to be statistically significant at age 40 years. As previously reported [13,30] attendance to the HIV screening program was predicted by a high CD4 count (> 350 cells/μL), which we again see as a proxy for being well-treated for one’s HIV infection. This causes further disparity, since women with low CD4 counts reportedly are at higher risk of ICC [3,16].

Predictors of attendance to screening programs in other studies were a diagnosis of HIV prior to pregnancy [7], previous pregnancies [7], history of abnormal cervical cytology [30], heterosexual transmission [35], screening advice from a gynaecologist rather than an infectious medicine specialist [30], older age [35], Black ethnicity [35] and integration of HIV care and ICC screening in the same setting [14].

In Denmark, the annual screening of WLWH is predominantly performed at the general practitioners. Contributing to the low coverage of cervical screening in WLWH most notably seen the first year after HIV diagnosis could be a combination of lack of knowledge and information from the health care professionals at the HIV centres to WLWH and lack of knowledge among general practitioners on the intensified HIV screening program for WLWH.

Only about a half of WLWH followed the general population screening program before HIV diagnosis. This screening behaviour is hard to interpret, since the group of WLWH followed before HIV diagnosis is smaller, time of follow-up varies and women immigrating to Denmark with HIV cannot be surveyed. Yet, we speculate whether this group of women generally practice less self-care than women in the general population.

Screening attendance for women in the general population was 83% in the end of the study period, which is a bit higher than the 3-year coverage reported for the entire eligible population of Denmark about 76% in 2010 [36].

The major strength of our study is the nationwide population-based design, linking the nationwide registers DHCS, CRS and DPDB, with very limited loss to follow-up [37], which ensures that data is not subject to recall bias. Moreover, our ability to integrate data on pregnancy, immigration, hysterectomy and prior ICC into the analyses optimizes accuracy of results.

Some limitations need to be considered: Baseline information on WLWH was those reported by the providers and then retrospectively summarized for this study. Additionally, we have not assessed dysplasia. A woman with a history of dysplasia might for a period due to gynaecological treatment and guidelines attend cervical screening more often and if rates of dysplasia are increased in WLWH in Denmark as seen in foreign cohorts [1,3,7-11], we might tend to overestimate adherence to the cervical screening program amongst WLWH.

We chose to evaluate adherence to the screening program on a monthly basis for all women during the study period. We did this to analyse the dependency on co-variables at specific arbitrarily chosen ages in order to have a single, easily defined, endpoint for each woman that could also reflect the development over age for the predictors in the model. Using this mode of analysis, we could model the times of cytology directly and allow for estimates that reflects coverage. The analysis was chosen over a more dynamic model that would rather reflect delay in screening behaviour than coverage. However, the chosen analysis does not provide the possibility of estimates of age as a predictor and represents a reduction of data. Finally, ethnicity of controls was not assessed in the analyses. We are far from complying with cervical screening guidelines for WLWH. Still, incidence of cervical dysplasia in WLWH in Denmark is unknown. However, if rates of dysplasia are high as reported from other cohorts [1,3,7-11], this yields for new approaches to cervical screening in WLWH. We support the idea of cytology as part of an annual review [15,33] and integration of HIV care and cervical screening in a single clinic setting [14,35,38]. Another measure-inspired by Australian health authorities [15] – could be an opt in automated reminder system with written invitations to women with overdue cervical cytologies. Moreover, targeted public health messages aimed at health care professionals at HIV centres, general practitioners and WLWH are essential.

## Conclusion

The majority of WLWH do not follow the recommended HIV screening program for ICC and they display lower attendance to the general population screening program than controls. This yields for new approaches to increase attendance to cervical screening in WLWH, since low ICC screening attendance may lead to overrepresentation of cervical dysplasia and ICC in WLWH in Denmark.

## Abbreviations

CI: Confidence interval; DHCS: Danish HIV cohort study; HAART: Highly active antiretroviral therapy; HPV: Human papillomavirus; IDU’s: Intravenous drug users; ICC: Invasive cervical cancer; IQR: Interquartile range; OR: Odds ratio; PIN: Personal identification number; CPR: The civil personal registration number; CRS: The civil registration system; DCR: The Danish cancer registry; DPDB: The Danish pathology data bank; NPR: The national patient registry; WLWH: Women living with HIV.

## Competing interests

KT has received research funding from Abbott and honoraria from Janssen-Cilag and GlaxoSmithKline/Viiv. TLK has received research funding from Roche, Bristol-Myers Squibb, Merck Sharp & Dohme, GlaxoSmithKline/Viiv, Abbott, Boehringer Ingelheim, Janssen-Cilag, and Swedish Orphan. JJ has received advisory board fees from Sanofi Pasteur MSD and consultant fees from Merck Denmark. AML has received research funding from Abbott and honoraria from Bristol-Myers Squibb, Merck Sharp & Dohme, GlaxoSmithKline, Boehringer Ingelheim and Janssen-Cilag. SL, SJF, IJ, GP, MH and MS report no conflicts of interest.

## Authors’ contributions

KT: Analyzed and interpreted data and drafted manuscript. SL: Biostatistician; Involved in analysis and interpretation of data and provided critical review of manuscript. SJF: Contributed to conception and study design and provided critical review of manuscript. Involved in analysis and interpretation of data. ISJ: Contributed to conception and study design and provided critical review of manuscript. TLK: Contributed to conception and study design and provided critical review of manuscript. GP: Contributed to conception and study design and provided critical review of manuscript. JJ: Contributed with knowledge on the DPDB and cervical screening in Denmark and provided critical review of manuscript. MH: Contributed to conception and study design and provided critical review of manuscript. Involved in analysis and interpretation of data. MS: Contributed to conception and study design and provided critical review of manuscript. AML: Principal investigator; Involved in conception and study design and provided critical review of manuscript. Involved in analysis and interpretation of data. All authors read and approved the final manuscript.

## Pre-publication history

The pre-publication history for this paper can be accessed here:

http://www.biomedcentral.com/1471-2334/14/256/prepub
